# Relieving lipid accumulation through UCP1 suppresses the progression of acute kidney injury by promoting the AMPK/ULK1/autophagy pathway

**DOI:** 10.7150/thno.56082

**Published:** 2021-03-04

**Authors:** Wei Xiong, Zhiyong Xiong, Anni Song, Chuntao Lei, Chen Ye, Chun Zhang

**Affiliations:** 1 Department of Nephrology, Union Hospital, Tongji Medical College, Huazhong University of Science and Technology, Wuhan 430022, China.; 2 Department of Urology, Union Hospital, Tongji Medical College, Huazhong University of Science and Technology, Wuhan 430022, China.

**Keywords:** lipids, UCP1, metabolic reprogramming, autophagy, acute kidney injury

## Abstract

**Rationale:** Acute kidney injury (AKI) is a serious clinical emergency with an acute onset, rapid progression, and poor prognosis. Recent evidence suggests that AKI is accompanied by significant metabolic abnormalities, including alterations in lipid metabolism. However, the specific changes in lipids in AKI, and their role and regulation mechanisms are currently unclear.

**Methods:** Quantitative metabolomics was performed in AKI models to reveal the differences of lipid metabolism-related products. Regulated pathway was detected by western blot, qRT-PCR, immunoblot analysis and immunohistochemistry.

**Results:** The present study systematically analyzes the changes in lipid composition in AKI for the first time and find that the degree of lipid accumulation was highly correlated with uncoupling protein 1 (UCP1). Importantly, relieving lipid accumulation in AKI by upregulating UCP1 can significantly inhibit the progression of AKI through promoting AMPK/ULK1/autophagy pathway.

**Conclusions:** The present findings suggest that lipid accumulation in AKI is directly regulated by UCP1, which can activate cell autophagy and thus significantly inhibit disease progression. It will provide new ideas and targets for the treatment of AKI.

## Introduction

Acute kidney injury (AKI) is a clinical syndrome characterized by a sharp decline in renal function over a short period of time (within 48 h) caused by damage to the structure or function of the kidney 1. Its clinical features include rapid progress, difficult early diagnosis, and high mortality 2. At present, treatment of AKI involves mainly symptomatic and supportive therapy, as well as renal replacement; however, their effect on AKI patients is very limited 3, 4. Therefore, clarifying the new mechanism of AKI and developing new methods for the early diagnosis and treatment of AKI have become the hotspots and difficulties of the current research.

In recent years, metabolic abnormalities have been found to be highly related to an increasing number of diseases, and are even considered to be a key factor in the occurrence and development of tumors, immunity, inflammation and other related diseases 5. Similarly, previous studies have confirmed that kidney diseases are highly related to metabolic abnormalities. It can not only promote the occurrence of kidney diseases, but also lead to their progression as a result 6, 7. These abnormal metabolisms include glucose metabolism, amino acid metabolism, and lipid metabolism abnormalities. However, currently, research on kidney diseases and metabolism mainly focuses on glucose metabolism in chronic kidney disease, and there are few systematic studies on lipid metabolism and AKI.

Uncoupling protein-1 (UCP1), located in the inner membrane of the mitochondria, is the most important marker of brown adipose tissue (BAT) 8. It promotes mitochondrial inner membrane proton conduction and decouples ATP synthesis from cellular respiration, and ultimately consume lipids to generate heat without additional ATP production 9. UCP1 is even considered to be a key factor in many metabolic diseases such as diabetes and obesity 10-12. Recently, studies have reported that UCP1 also played a critical role in the lipid metabolism of renal clear cell carcinoma (ccRCC). It could promote tumor cell “slimming”, effectively eliminate lipids accumulation in ccRCC and inhibit its progression 13, 14. However, UCP1-mediated lipid consumption has rarely been studied in renal non-neoplastic diseases.

Autophagy is an important process for the maintenance of intracellular homeostasis as it facilitates the transport of damaged, denatured, or aged proteins and organelles into lysosomes for degradation 15. It acts as a double-edged sword, which promotes apoptosis by activating type II programmed death, while at the same time delaying the onset of apoptosis and protecting cells from apoptosis or necrosis 16-18. Autophagy has been shown to play an important role in AKI, which could significantly inhibit apoptosis, thus delaying AKI progression 19-21. It is also highly related to lipid metabolism, and has been proved to be regulated by lipid droplets to help maintain endoplasmic reticulum homeostasis 22. However, little is known about the exact relationship between autophagy and lipid metabolism in AKI.

In this study, we systematically analyzed the difference in lipid composition in AKI, discovered the phenomenon of lipid accumulation in AKI, and clarified its correlation with UCP1 for the first time. Relieving lipid accumulation through UCP1 could suppress the progression of AKI by promoting AMPK/ULK1/Autophagy pathway.

## Methods

### Animals

All animal experiments in this study were carried out in accordance with the Guidelines for the Use and Care of Laboratory Animals of the National Institutes of Health and were approved by the Animal Care and Use Committee of Tongji Medical College. The accreditation number of the investigator is TY20160614 assigned by Hubei Provincial Laboratory Animal Public Service Center. Adult C57BL/6 mice, Sprague-Dawley rats, and Wistar rats were provided by Charles River (Beijing, China). All mice had free access to water and standard chow. Male C57BL/6 mice (weighing 20-25 g, aged 6-8 weeks) were injected with cisplatin (25 mg/kg, Sigma-Aldrich, St Louis, MO, USA) intraperitoneally and euthanized at days 1, 2, and 4 after the injection. The control group was given the same volume of saline. The time point of cisplatin stimulation for 2 days was selected as the time point of follow-up intervention experiments. Mice were euthanized two days after cisplatin injection. Prior to removing the kidneys, the animals were anesthetized with sodium pentobarbital. The renal cortex, medulla, and papilla were dissected at 4 °C. The glomeruli were separated by the sieving method, whereby the kidneys were flushed with ice-cold Krebs-Henseleit-saline buffer through the aortic catheter. Using the same buffer, the minced renal cortex was passed through three steel sieves (200, 120, and 80 μm), the glomeruli were recovered from the 80 μm sieve, washed, and resuspended. The renal tubular cells were separated by pressing the fragments of the renal cortex through an 80 μm sieve and snap-frozen in liquid nitrogen and stored at -80 °C until total RNA or protein was extracted. The animals were euthanized after 7 days of continuous injection of CL316243 (Tocris Bioscience, Bristol, UK) or saline via the caudal vein (2 mg kg^-1^ d^-1^) and intraperitoneally (2 mg kg^-1^ d^-1^). Cisplatin injection was performed 5 days after CL316243 or saline injection. The specific operation method was shown in **Figure S7B**. Blood samples of mice were collected and placed at room temperature for 2 h, then centrifuged at 4000 rpm at 4 °C for 15 min. The supernatant was taken and detected by AutoChemistry Analyzer (DIRUI, Jilin, China) to assess serum creatinine (SCr) and blood urea nitrogen (BUN).

### Intrarenal adenovirus delivery

UCP1 overexpression adenovirus and control adenovirus were obtained from Vigenebio (Shandong, China). Each of them was delivered into the mouse kidney by intraparenchymal injection 7 days prior to establishment of the AKI model. Before the injection, the mice were temporarily anesthetized and the abdominal cavity was opened to expose the bilateral kidneys. Then, 100 μL of UCP1 or control adenovirus (1 × 10^11^ pfu mL^-1^) was aspirated with a 31G needle. Three to five sites were selected from each kidney to slowly inject the virus into the renal cortex. A total of 200 μL of virus was injected into both kidneys. The specific operation method was shown in **Figure S7A**.

### Immunohistochemical and immunofluorescent staining

4-μm formalin-fixed paraffin-embedded sections were subjected to immunohistochemical staining. For antigen retrieval, the sections were deparaffinized and rehydrated, incubated in EDTA at 120 °C for 5 min, and then with 3% H_2_O_2_ for 15 min at room temperature. The sections were blocked with fetal calf serum. The slides were incubated with an anti-UCP1 (ab10983, 1:100; Abcam, Cambridge, UK) or anti-LC3 (14600, 1:100; Proteintech, Manchester, UK) antibody overnight at 4 °C. The sections were then washed in phosphate-buffered saline and incubated with biotinylated goat anti-rabbit antibody (Beyotime, Jiangsu, China) for 20 min. After being stained with hematoxylin, the sections were dehydrated with an alcohol gradient, sealed with neutral gum, and observed under a light microscope.

For immunofluorescent staining, fresh tissues were frozen. Tissues and cells were fixed in 4% paraformaldehyde, permeabilized with 0.5% Triton X-100 for 10 min, and then blocked with 5% goat serum. They were then incubated with the same primary antibodies as above (UCP1, 1:250; LC3, 1:250) as well as an anti-CD68 antibody (ab955, 1:250; Abcam). Alexa Fluor 488-conjugated donkey anti-rabbit IgG (H+L) (AS035, 1:250; ABclonal, Woburn, MA, USA) was used as the secondary antibody. Nuclei were stained with 4,6-diamidino-2-phenylindole (DAPI).

The apoptotic cells in cells and tissues were evaluated by the situ Apoptosis Detection kit (Roche, Mannheim, Germany). The experimental steps referred to the manufacturer's instructions. Each sample was randomly counted for 4 fields. The average value of apoptotic cells in these four fields was calculated. Then t test was used to compare the relative average values of different groups of samples. The computing method of CD68 positive cells was that each sample was randomly counted for 4 fields. The average value of CD68 positive cells in these four fields was calculated. Then t test was used to compare the average values of different groups of samples.

For BODIPY dye, after the frozen section was slightly dried, the histochemical pen was used to draw a circle around the tissue, and the BODIPY (131083-16-4, 1:500; B&P Biotech, Hangzhou, China) staining solution was dripped in the circle, and incubated at room temperature in dark for 60 min. Then the slides were placed in PBS (pH7.4) and washed on the decolorizing shaker for 3 times, 5 min each time. After the slices were slightly dried, DAPI staining solution was dripped in the circle and incubated at room temperature for 10 min. The slides were placed in PBS (pH7.4), shaken and washed on the decolorizing shaking table for 3 times, 5 min each time. After a little drying, the sections were sealed with anti-fluorescence quenching sealing agent. Sections were observed under fluorescence microscope and images were collected.

### Cell culture, treatment and transfection

The HK2 cell line was obtained from the American Type Culture Collection (Manassas, VA, USA). Cells were cultured at 37 °C and 5% CO_2_ in MEM supplemented with 10% fetal bovine serum and 1% penicillin-streptomycin in an incubator. Cells were treated with cisplatin (20 µM; Sigma-Aldrich) for 3-24 h, with 12 h being selected as the stimulation time for subsequent experiments. Cells were collected after stimulation with cisplatin for 12 h. CL316243 (Tocris Bioscience) was administered to cells at a concentration of 1 μM for 48 h before cells collection and 12 h before cisplatin treatment. Chloroquine was purchased from MCE (Monmouth Junction, NJ, USA) and incubated the cells for 12 h at a concentration of 50 μM before cells collection, that is, the cells were incubated with cisplatin at the same time. Oil red O was obtained from Wuhan Servicebio technology (Wuhan, China). The lentiviruses expressing UCP1 and the corresponding control vector were obtained from Genechem (Shanghai, China) and were transfected as specified by the manufacturer.

### Western Blotting assays

The collected tissues and cells were lysed with RIPA lysis buffer (Beyotime) to extract the proteins. Next, 50 μg of protein was separated by sodium dodecyl sulfate-polyacrylamide gel electrophoresis followed by transfer to polyvinylidene difluoride membranes (Millipore Corp., Bedford, MA, USA). The membranes were blocked in 5% non-fat dried skimmed milk (Beyotime) for 1 h at room temperature and incubated with primary antibodies overnight at 4 °C. Finally, the membranes were incubated in blocking buffer with secondary antibody (1:2500; AntGene, Wuhan, China) for 2 h before detection. Glyceraldehyde-3-phosphate dehydrogenase (GAPDH) was used as internal control. Primary antibodies against the following targets were employed: UCP1 (ab10983, 1:1000), GAPDH (60004-1-Ig, 1:1000; Proteintech), NGAL (ab63929, 1:1000; Abcam), Bax (50599-2-Ig, 1:1000; Proteintech), Bcl-2 (12789-1-AP, 1:1000; Proteintech), cleaved caspase-3 (9664, 1:1000; Cell Signal Technology, Danvers, MA, USA), P-AMPK (AP0116, 1:1000; ABclonal), AMPK (A17290, 1:1000; ABclonal), P-ULK1 (AP0736, 1:1000; ABclonal), ULK1 (A8529, 1:1000; ABclonal), and P62 (A11250, 1:1000; ABclonal). The densitometric analysis of western blot images was performed by ImageJ2x. Statistical analyses were performed using SPSS Statistics 22.0 (IBM SPSS, Chicago, IL) and Excel 2016 (Microsoft).

### RNA isolation and real-time PCR analysis

TRIzol reagent (Takara, Dalian, China) was used to extract RNA from tissues and cells, which was then reverse-transcribed into cDNA using the PrimeScriptRT Master Mix (Takara) according to the manufacturer's instructions. A NanoDrop 2000 spectrophotometer (NanoDrop Technologies, Wilmington, DE, USA) was used to measure mRNA levels of the target genes. Quantitative PCR analysis was conducted with SYBR Green mix (Thermo-Scientific, Waltham, MA, USA). Samples were normalized using GAPDH. The primer sequences were as follows:

### Lipidomics sequencing

Fresh renal cortical tissues of control mice and mice treated with cisplatin for 4 days were collected and immediately stored in liquid nitrogen for lipidomics sequencing. Lipidomics sequencing was provided by Novogene Co. Ltd (Nanjing, China). Transfer the liquid sample (100 μL) into a glass tube, then add 900 μL water, 2 ml methanol and 0.9 ml dichloromethane, and mix well. After adding 1.0 ml water and 0.9 ml dichloromethane, the sample was rotated to emulsification and centrifuged at 3000 rpm at 4 °C for 5 min. The lower dichloromethane layer is then transferred to a fresh glass tube with a glass syringe. 2 ml dichloromethane was added to the supernatant and centrifuged at 3000 rpm at 4 °C for 5 min. The collected lower dichloromethane layers are combined and rotated in a vacuum concentrator until dry. The dried pellets were redissolved in 500 μL methanol/dichloromethane and analyzed by LC-MS/MS. Waters ACQUITY UPLC I-Class system was used for chromatographic analysis. The sample was injected into a C18 CSH column (100 mm × 2.1 mm, 1.7 μm; Waters) with a 20 min linear gradient and a flow rate of 0.4 ml/min. The column temperature was set at 45 °C. Mobile phase buffer A was acetonitrile/water (1/4), containing 10 mM ammonium formate and 0.1% formic acid, while buffer B was acetonitrile/isopropanol (1/9), containing 10 mM ammonium formate and 0.1% formic acid. The solvent gradient was set as follows: 40% B, initial; 43% B, 2 min; 50% B, 2.1 min; 54% B, 12 min; 70% B, 12.1 min; 99% B, 18 min; 40% B, rebalance for 2 min. After separation by UPLC, mass spectrometry was carried out using Xevo G2-S Q-TOF and electrospray ionization (ESI) source (Waters, Manchester, UK). The dynamic range of Xevo G2-S Q-TOF mass spectrometry was enhanced to improve the isotopic distribution and mass accuracy and reduce the high ionic strength. In positive ion mode, MS parameters were as follows: capillary voltage is set to 2.5 kV, cone voltage was set to 24 V, source temperature was set to 100 °C, desolvent temperature was set to 400 °C, desolvent gas flow rate was set to 800 L/h, cone gas flow rate was set to 50 L/h. Acquisition was performed from m/z 100 to 1500. In negative ion mode, MS parameters were as follows: capillary voltage was set at 2.5 kV, cone voltage was set at 25 V, source temperature was 100 °C, desolvent temperature was 500 °C, desolvent gas flow rate was 600 L/h, cone gas flow rate was 10 L/h. Acquisition was performed from m/z 100 to 1500. The original data were imported into the Progenesis QI (Waters) for peak alignment to obtain a list of peaks containing the retention time, m/z and peak area of each sample. Database including Lipidmaps (http://www.lipidmaps.org), HMDB database (http://www.hmdb.ca), NIST (https://chemdata.nist.gov). The internal lipid database was used to identify the metabolites. The mass error used was 5 ppm.

KEGG (Kyoto Encyclopedia of Genes and Genomes) is the main public database about pathway (http://www.genome.jp/kegg/). Pathway analysis can identify the most important biochemical metabolic pathway and signal transduction pathway involved in metabolites. The enrichment results were based on KEGG pathway. Hypergeometric test was applied to find out the enriched pathways in different metabolites compared with the background of all identified metabolites, as shown in the figure below.


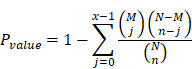


Where N was the number of metabolites with KEGG annotation information in all metabolites, n was the number of differential metabolisms in N, M was the number of metabolites annotated to a KEGG entry in all metabolites, and X was the number of differential metabolites annotated to a KEGG entry. The p-value was calculated, and the KEGG term satisfying this condition was defined as the KEGG term significantly enriched in the different metabolites with p-value ≤0.05 as the threshold. KEGG significance analysis can determine the main biological functions of different metabolites.

### Oil Red Staining

#### Cell

The cells were cultured in 6-well plates to 30% fusion. The oil red dye was prepared with the ratio of 2:3 saturated oil red and ultrapure water. The cell culture medium was removed and the cells were washed twice with PBS. 4% paraformaldehyde was used to fix cells, then was removed, and made the sample dry naturally after 10 min. The prepared oil red fuel was added and the sample was dyed at room temperature for 30 min. Then the excess oil red fuel was removed with PBS, and the sample was dried and observed with microscope.

#### Animal tissue

The cryopreserved slices were heated at room temperature, soaked in distilled water and washed out the embedding agent. Then the slices were soaked in 60% isopropanol for 2 min and stained with oil red dye for 2-5 min. The slices were colored with 60% isopropanol and washed immediately after color mixing. Hematoxylin was re-stained for 1 min, and the differentiation was induced by hydrochloric acid and alcohol for 1-5 s. Glycerin gelatin was sealed and the sample was observed under microscope.

### Triglyceride detection

Triglyceride assay kit (A110-1-1) was bought from Nanjing Jiancheng Bioengineering Institute. The tissue was homogenized and then pyrolyzed with 200 μL 2% Triton X-100 for 40 min. Then 2.5 μL of the above product was added into 250 μL working solution, incubated at 37 °C for 10 min, and some samples were taken for protein quantification. Finally, the detection was carried out with a microplate reader at the wavelength of 510 nm. The calculation formula is as follows:


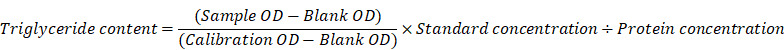


### Tubule damage assessment

The kidney tissues were cut into small pieces, dehydrated and embedded in paraffin, then made into paraffin embedded blocks, sliced and stained with HE. The pathological sections were observed under 400× light microscope. Five fields were randomly selected, and 10 renal tubules were selected for each field. The scoring criteria were Paller's method, i.e. obvious expansion of renal tubules, flattened cells (1 point), tubular type (2 points), exfoliated and necrotic cells in the lumen of renal tubules, but no tubular type and cell debris (1 point), granular degeneration of epithelial cells (1 point), vacuolar degeneration (1 point), nuclear pyknosis (1 point). The degree of injury was judged by the score. SPSS statistical software was used to analyze the Paller score of HE sections in each group, and t test was used to evaluate the significance of the difference between the two groups.

### Transmission electron microscopy

The cells were collected by centrifugation. The medium was removed and the electron microscope fixative was added. After pre-embedding, agarose blocks with samples were fixed with 1% OsO4 in 0.1 M PB (pH 7.4) for 2 h at room temperature. The tissues were dehydrated with 30% - 50% - 70% - 80% - 95% - 100% alcohol for 20 min each time and 100% acetone for 15 min each time. The embedding plate was polymerized in 60 °C oven for 48 h, and then sliced in 60-80 nm ultra-thin microtome. The 150-mesh copper was used to remove the slices. The copper mesh was dyed in 2% uranyl acetate saturated alcohol solution for 8 min in dark. 2.6% lead citrate solution for 8 min in dark. The copper mesh slices were put into the copper mesh box and dried overnight at room temperature. The images were collected and analyzed under transmission electron microscope. Autophagy was considered to be crescent or cup-shaped, bilayer or multilayered, and contain the cytoplasm.

### Statistical analysis

All data were expressed as mean ± SEM. A t-test was used for the comparison of two groups and one-way analysis of variance (ANOVA) was used for more than two groups. The significance value was determined as P < 0.05.

## Results

### AKI is accompanied by significant accumulation of lipids and is highly positively correlated with the severity of kidney injury

**Figure 1:**
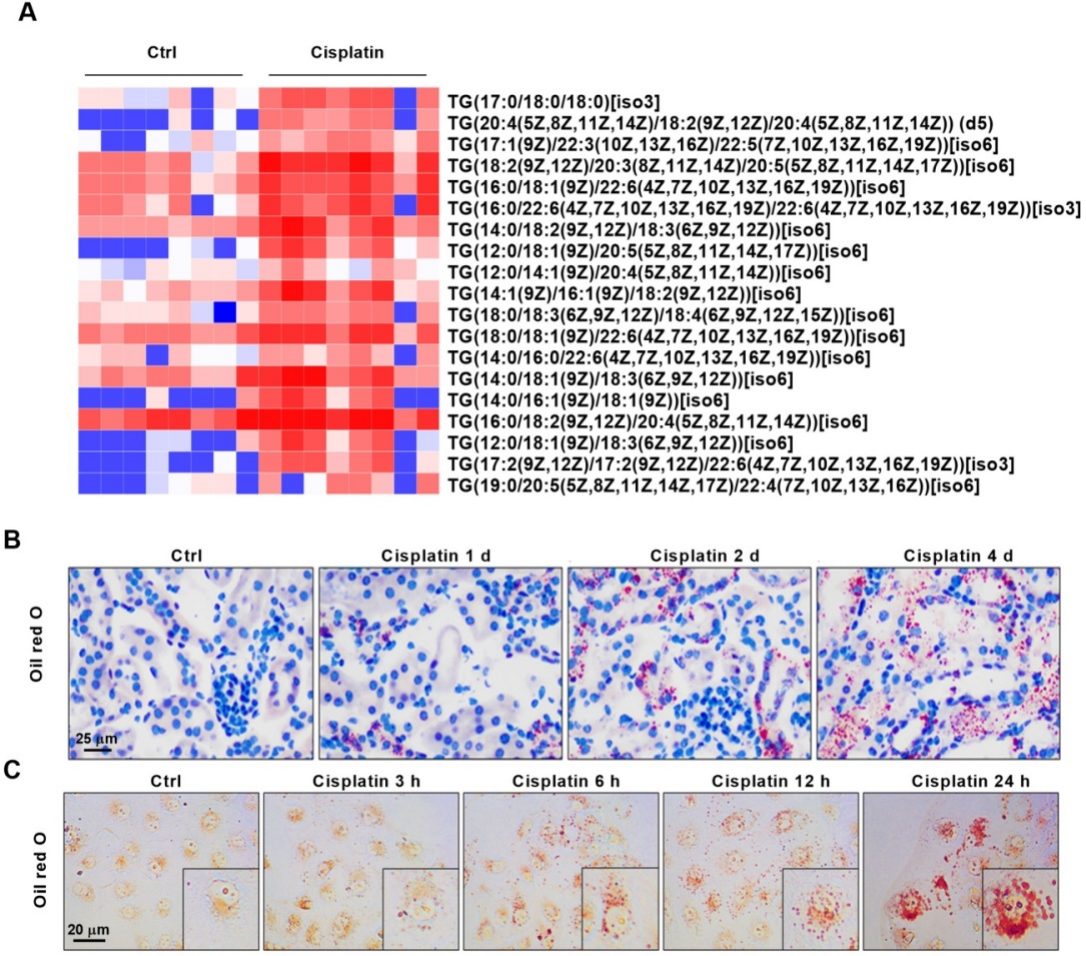
** AKI is accompanied by significant accumulation of lipids and correlates positively with the severity of kidney injury.** (A) Heat map showing differential expression of TGs based on lipid metabolomics results (n = 8). (B) Photomicrographs showing oil red O staining of control and cisplatin-treated mice on days 1, 2, and 4. (C) Photomicrographs showing oil red O staining of HK2 cells exposed to cisplatin for 3, 6, 12, and 24 h. ****p < 0.0001, ***p < 0.001, **p < 0.01, *p < 0.05 vs. control.

We performed principal component analysis (PCA) on the fresh kidney tissues of control mice and cisplatin-stimulated mice, and the results showed that the two sample models were stable and reliable (**Figure S1**). Quantitative metabolomics of AKI models revealed for the first time the differences of lipid metabolism-related products in renal cortex between cisplatin-induced AKI mice and control mice (**Figure S2-S3**). Based on lipid metabolomics analysis results, we found a significant increase in the content of various types of triglycerides (TGs) in AKI tissues (**Figure 1A**). Enrichment analysis of Kyoto Encyclopedia of Genes and Genomes (KEGG)-related pathways revealed that differentially abundant compounds correlated with specific metabolic pathways and particularly the lipid metabolic pathway (**Figure S4**). Given the observed lipid accumulation in AKI, experiments were carried out to determine its clinical relevance. Oil red O staining was performed on AKI animal specimens characterized by different severity of the disease. The results showed that lipid deposition in the renal tissue of mice gradually increased with the extension of disease progression (**Figure 1B, Figure S5A**-**B**). An analogous result was found *in vitro*, whereby the degree of lipid accumulation increased with the severity of cell damage (**Figure 1C, Figure S5C**). These meant that lipid accumulation in AKI was positively correlated with the severity of the disease.

### UCP1 is significantly downregulated in AKI and is highly negatively correlated with the severity of kidney injury

**Figure 2:**
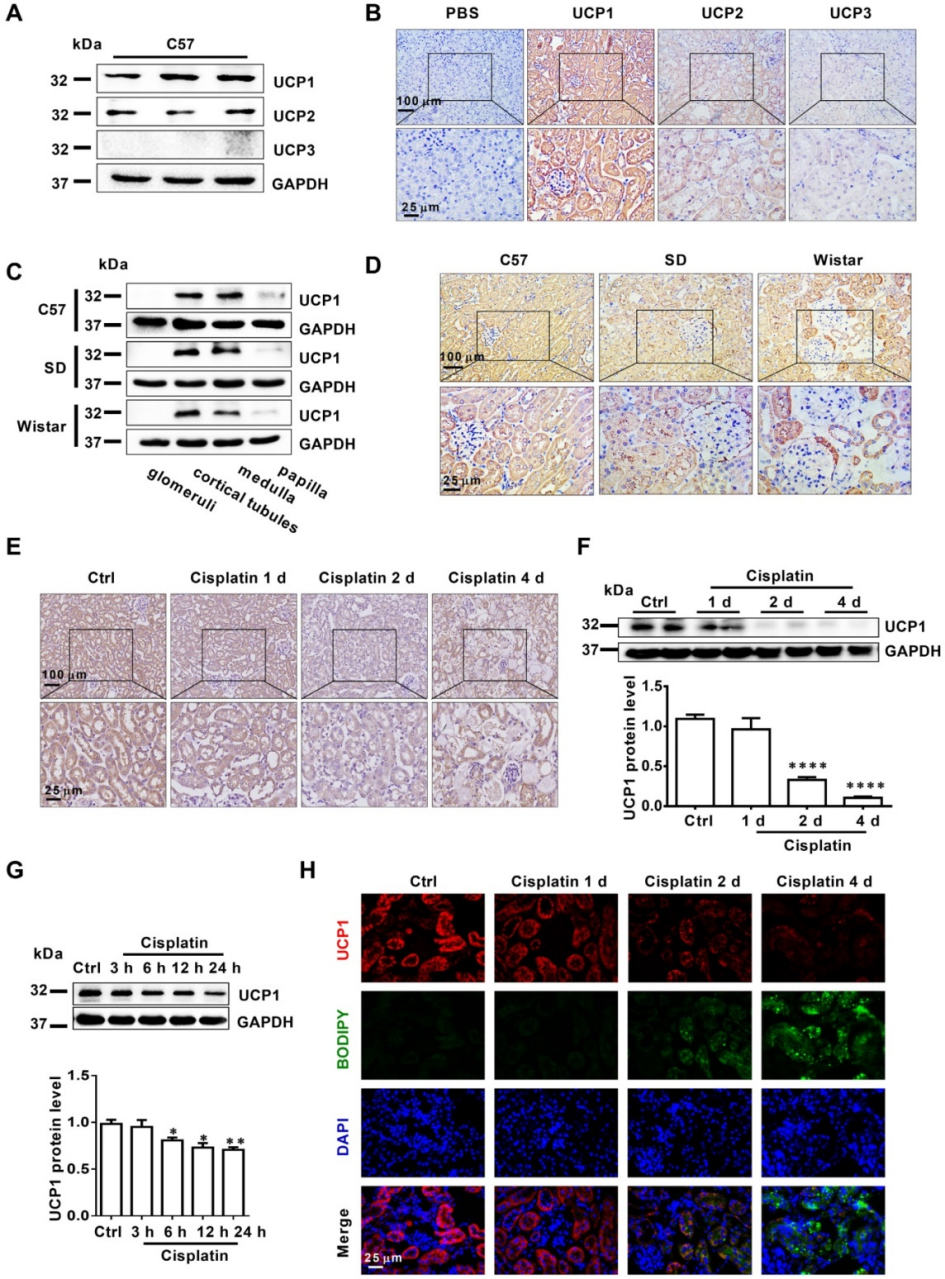
** UCP1 is significantly downregulated in AKI and correlates negatively with the severity of kidney injury.** (A) Representative western blot images showing the levels of UCPs in the kidneys of C57 mice (n = 3). (B) Representative photomicrographs showing immunohistochemical staining against UCPs in the kidneys of C57 mice. The negative control employed phosphate-buffered saline (PBS) instead of antibody. (C) Representative western blot images showing the expression of UCP1 in different sections of the kidney from C57 mice and Sprague-Dawley (SD) and Wistar rats (n = 3). (D) Representative photomicrographs showing immunohistochemical staining against UCP1 in different sections of the kidney from C57 mice and Sprague-Dawley (SD) and Wistar rats. (E) Photomicrographs showing immunohistochemical staining against UCP1 in AKI animal models induced with cisplatin for 1, 2, and 4 days. (F) Western blot images and corresponding quantifications of UCP1 in cisplatin-induced AKI animal models (n = 5). (G) Western blot images and corresponding quantifications of UCP1 in HK2 cells exposed to cisplatin for 3, 6, 12, and 24 h. (H) Confocal microscopic images of UCP1 and BODIPY dye in the kidneys of mice treated with cisplatin for 1, 2, and 4 days. Nuclei are counterstained with DAPI. ****p < 0.0001, ***p < 0.001, **p < 0.01, *p < 0.05 vs. control.

The superfamily of uncoupling proteins (UCPs) is closely related to energy metabolism and mediates lipid consumption in several diseases 10, 23. Based on the function of UCPs, we characterized the expression of UCP1, UCP2 and UCP3 in normal C57 mice renal tissues by western blot and immunohistochemistry. The results showed that UCP1 and UCP2 were well expressed, whereas UCP3 was hardly detected (**Figure 2A**-**B**). Among UCPs, UCP1 is considered to be the most critical gene for lipid degradation while UCP2 has no direct correlation to it 24, 25. Hence, UCP1 was chosen for further analyses, which confirmed its high expression in cortical tubules, some expression in the medulla, and almost no expression in the glomeruli and papilla of C57 mice, Sprague-Dawley rats, and Wistar rats (**Figure 2C**-**D**). In order to further confirm the expression site of UCP1 in kidney, we used lectin dye to show the morphology of kidney and then stained UCP1. The results showed that UCP1 was mainly expressed in renal tubules (**Figure S6A**). AQP1, mainly expressed in renal proximal tubules, and UCP1 had obvious co-localization (**Figure S6B**). The above results suggested that UCP1 might play a potentially important role in the structure and function of renal tubules.

To explore the correlation between UCP1 and AKI, we tested the expression of UCP1 in AKI models both *in vivo* and *in vitro*. The results showed that UCP1 was significantly down-regulated in AKI mice and, even more importantly, its expression gradually decreased with the aggravation of kidney injury (**Figure 2E**-**F**). *In vitro*, the expression of UCP1 in HK2 cells decreased with the prolongation of cisplatin stimulation time (**Figure 2G**). These findings indicated that UCP1 correlated negatively with the severity of AKI. Given the strong correlation between UCP1 and lipid metabolism, we investigated the relationship between UCP1 and lipid accumulation as well. As shown in **Figure S6C**, lipid droplets were also co-localization with AQP1 as UCP1. Lipid-specific immunofluorescence and oil red O staining showed that the degree of lipid accumulation in AKI correlated negatively with UCP1 expression; whereby, as the degree of renal cell damage in AKI increased, while lipids accumulated, UCP1 expression gradually decreased (**Figure 2H**). This result suggested that UCP1 expression in AKI might affect lipid accumulation.

### Lipid accumulation in AKI is highly negatively correlated with UCP1

**Figure 3:**
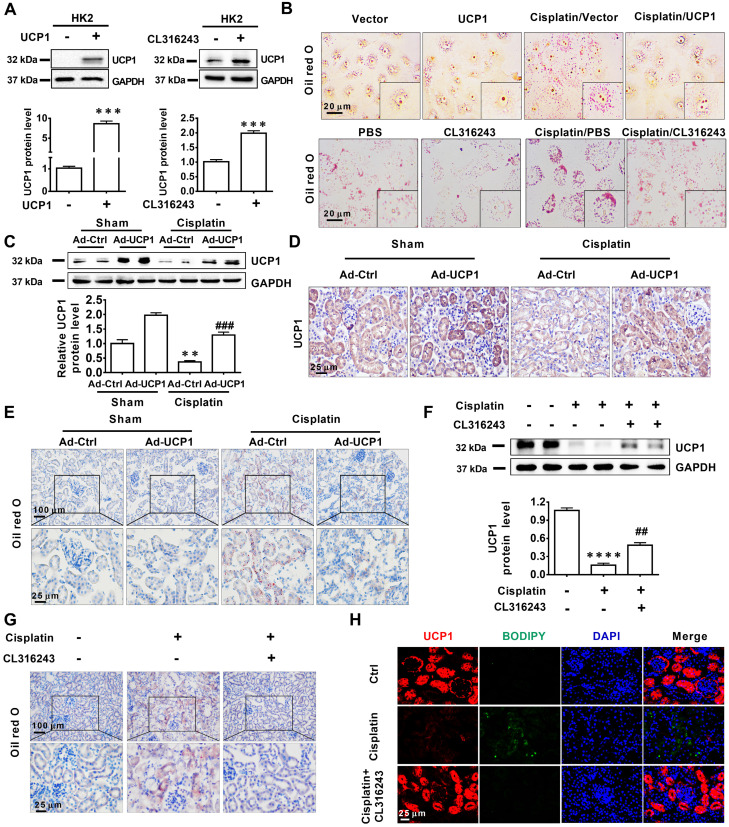
** Lipid accumulation in AKI is highly negatively dependent on UCP1.** (A) Western blot images and corresponding quantifications of UCP1 in HK2 cells overexpressing or not UCP1 (by way of lentivirus or agonist CL316243). (B) Photomicrographs showing oil red O staining of HK2 cells exposed to cisplatin with or without UCP1 overexpression. (C) Western blot images and corresponding quantifications of UCP1 in the kidney of C57 mice injected with control or UCP1-expressing adenovirus (n = 5). (D) Immunohistochemistry staining against UCP1 in AKI animal models specified in C. (E) Photomicrographs showing oil red O staining of kidneys in AKI models specified in C. (F) Western blot images and corresponding quantifications of UCP1 in AKI animal models treated with CL316243 (n = 5). (G) Photomicrographs showing oil red O staining of kidneys in AKI animal models treated with CL316243. (H) Representative confocal microscopic images of UCP1 and BODIPY dye in the kidneys of AKI animal models treated with CL316243. Nuclei are counterstained with DAPI. ****p < 0.0001, ***p < 0.001, **p < 0.01, *p < 0.05 vs. control; # vs. cisplatin.

Having established that UCP1 correlated negatively with lipid accumulation in AKI, we tried to clarify the underlying relationship. First, we used a UCP1-specific overexpression lentivirus and UCP1 agonist CL316243 to construct a cell model with upregulated UCP1 (**Figure 3A**). As shown in **Figure 3B**, lipid accumulation in HK2 exposed to cisplatin was significantly reduced by the overexpression of UCP1. Similar results were obtained with the UCP1 agonist CL316243. These findings suggested that UCP1 was involved in regulating lipid accumulation in AKI. In order to further verify this regulatory relationship and improve the reliability of the evidence, we employed an animal model of AKI overexpressing UCP1 by using UCP1 specific overexpression of adenovirus by renal multipoint injection (**Figure 3C**-**D**). Oil red O staining showed that overexpression of UCP1 could significantly alleviate lipid accumulation in AKI animal models (**Figure 3E**). In addition, TG kit was used to quantitatively assess lipid content in animal models. Similar results could be found that overexpression of UCP1 significantly reduced lipid accumulation in animal models of AKI (**Figure S7C**). Finally, the same results were observed also by using the UCP1 agonist CL316243 to induce UCP1 overexpression in an AKI animal model (**Figure 3F**-**H, Figure S7D**). Hence, all evidence indicated that, as the expression of UCP1 increased, lipid content in the AKI model decreased significantly.

### Upregulating UCP1 to relieve lipid accumulation during AKI can significantly inhibit disease progression by affecting inflammation and apoptosis *in vitro*

**Figure 4:**
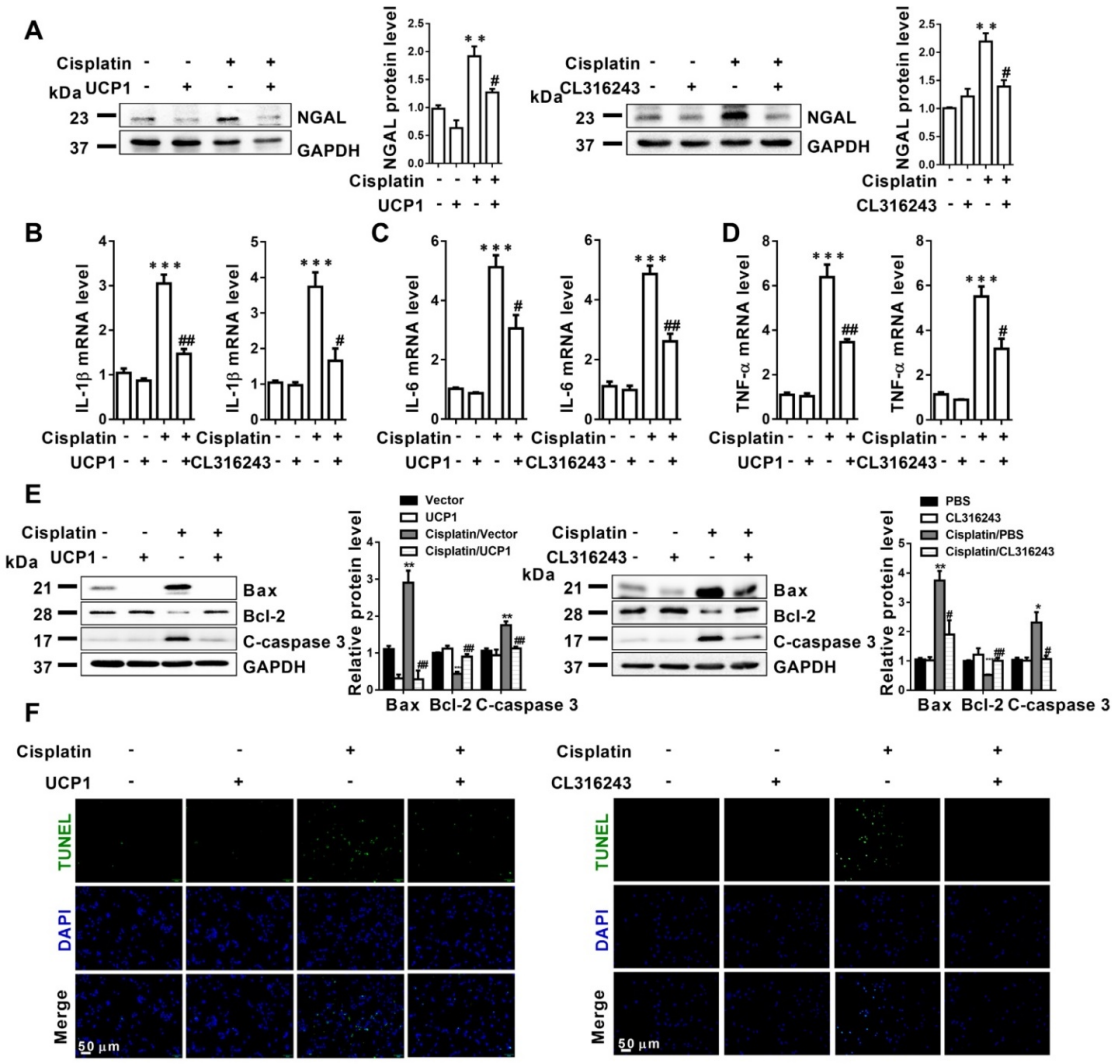
** Upregulation of UCP1 to relieve lipid accumulation in AKI significantly inhibits disease progression by affecting inflammation and apoptosis *in vitro*.** (A) Western blot images and corresponding quantifications of NGAL in HK2 cells exposed to cisplatin with or without UCP1 overexpression. (B-D) IL-1β, IL-6, and TNF-α mRNA levels in HK2 cells exposed to cisplatin with or without UCP1 overexpression. (E) Western blot images and corresponding quantifications of Bax, Bcl-2, and cleaved caspase-3 in HK2 cells exposed to cisplatin with or without UCP1 overexpression. (F) TUNEL assay fluorescence images of HK2 cells exposed to cisplatin with or without UCP1 overexpression. Nuclei are counterstained with DAPI. ****p < 0.0001, ***p < 0.001, **p < 0.01, *p < 0.05 vs. control; # vs. cisplatin.

Accumulated evidence pointed to significant lipid accumulation and abnormal expression of UCP1 in AKI, as well as strong correlation with the severity of kidney injury. To determine whether lipid accumulation impacted cellular function during AKI, we first used UCP1 overexpression lentivirus and UCP1 agonist CL316243 to construct a lipid accumulation clearance in AKI cell models. As shown in **Figure 4A**, upregulation of UCP1 to clear lipid accumulation in AKI caused significant downregulation of the renal tubular damage index, neutrophil gelatinase-associated lipocalin (NGAL), which indicated that clearing lipid accumulation by UCP1 could significantly alleviate the damage of model cells. Given that inflammation and apoptosis are the most important and direct causes of cell damage in AKI 26, 27, we quantified inflammation and apoptosis markers in the above cell models. Inflammation indicators interleukin (IL)-1β, IL-6, and tumor necrosis factor-alpha (TNF-α) exhibited a significant decrease with the upregulation of UCP1 (**Figure 4B**-**D**). A similar trend was observed for the apoptosis indicators Bax and cleaved caspase-3 (C-caspase 3), whereas Bcl-2 was increased (**Figure 4E**). Finally, direct quantitative assessment of cell apoptosis by terminal deoxynucleotidyl transferase dUTP nick end labeling (TUNEL) fluorescent staining confirmed that upregulating UCP1 alleviated apoptosis in AKI model cells (**Figure 4F**).

### Upregulating UCP1 to relieve lipid accumulation during AKI can significantly inhibit disease progression by affecting inflammation and apoptosis *in vivo*

**Figure 5:**
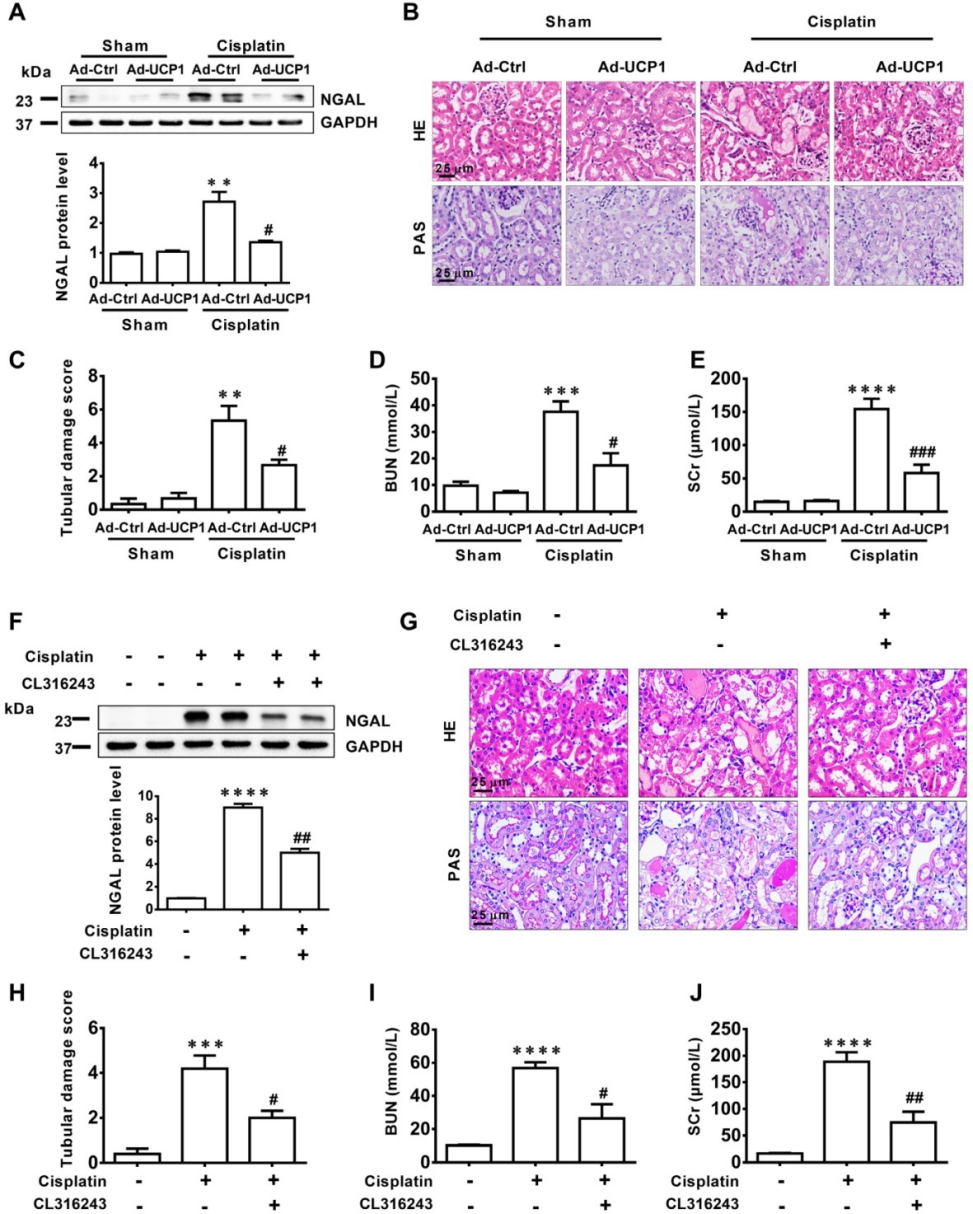
** Upregulation of UCP1 to relieve lipid accumulation in AKI significantly inhibits disease progression *in vivo*.** (A) Western blot images and corresponding quantifications of NGAL in AKI animal models induced by cisplatin and injected with control or UCP1-expressing adenovirus. (B, C) Representative photomicrographs and quantitative assessment of tubular damage showing the morphology of kidneys from AKI animal models specified in A. HE, hematoxylin & eosin; PAS, periodic acid Schiff staining. (D, E) BUN and SCr levels from AKI animal models specified in A (n = 5). (F) Western blot images and corresponding quantifications of NGAL in AKI animal models induced by cisplatin and treated with CL316243 (n = 5). (G, H) Representative photomicrographs and quantitative assessment of tubular damage showing the morphology of kidneys from AKI animal models specified in F. (I, J) BUN and SCr levels from AKI animal models specified in F (n = 5). ****p < 0.0001, ***p < 0.001, **p < 0.01, *p < 0.05 vs. control; # vs. cisplatin.

To verify the effect of lipids on the function of AKI *in vivo*, we constructed a UCP1 overexpression animal model by multi-point injection of UCP1 overexpressing adenovirus in the kidney to eliminate lipid accumulation in AKI. The renal damage indicator NGAL was markedly decreased in the UCP1 overexpression animal model (**Figure 5A**). Hematoxylin (HE) and periodic acid Schiff (PAS) staining revealed great improvement in renal morphology and the renal tubular damage score indicated a strong reduction in the degree of renal tubular damage after the increase in UCP1 mediated lipid consumption (**Figure 5B**-**C**). After stimulation with cisplatin, the renal tubules showed vacuolar degeneration, flattened cells and dilated lumen, while the overexpression of UCP1 significantly improved the pathological changes. A similar significant decrease was observed for blood serum creatinine (SCr) and urea nitrogen (BUN), two indicators of kidney damage (**Figure 5D**-**E**). The above results were further verified using the UCP1 agonist CL316243 in AKI animal model for lipid accumulation relief. NGAL was greatly decreased after the treatment (**Figure 5F**), while kidney morphology, renal tubular injury score, SCr, and BUN were all significantly improved (**Figure 5G**-**J**). Therefore, *in vivo*, we can also conclude that upregulating UCP1 to relieve AKI lipid accumulation can inhibit AKI progression.

**Figure 6:**
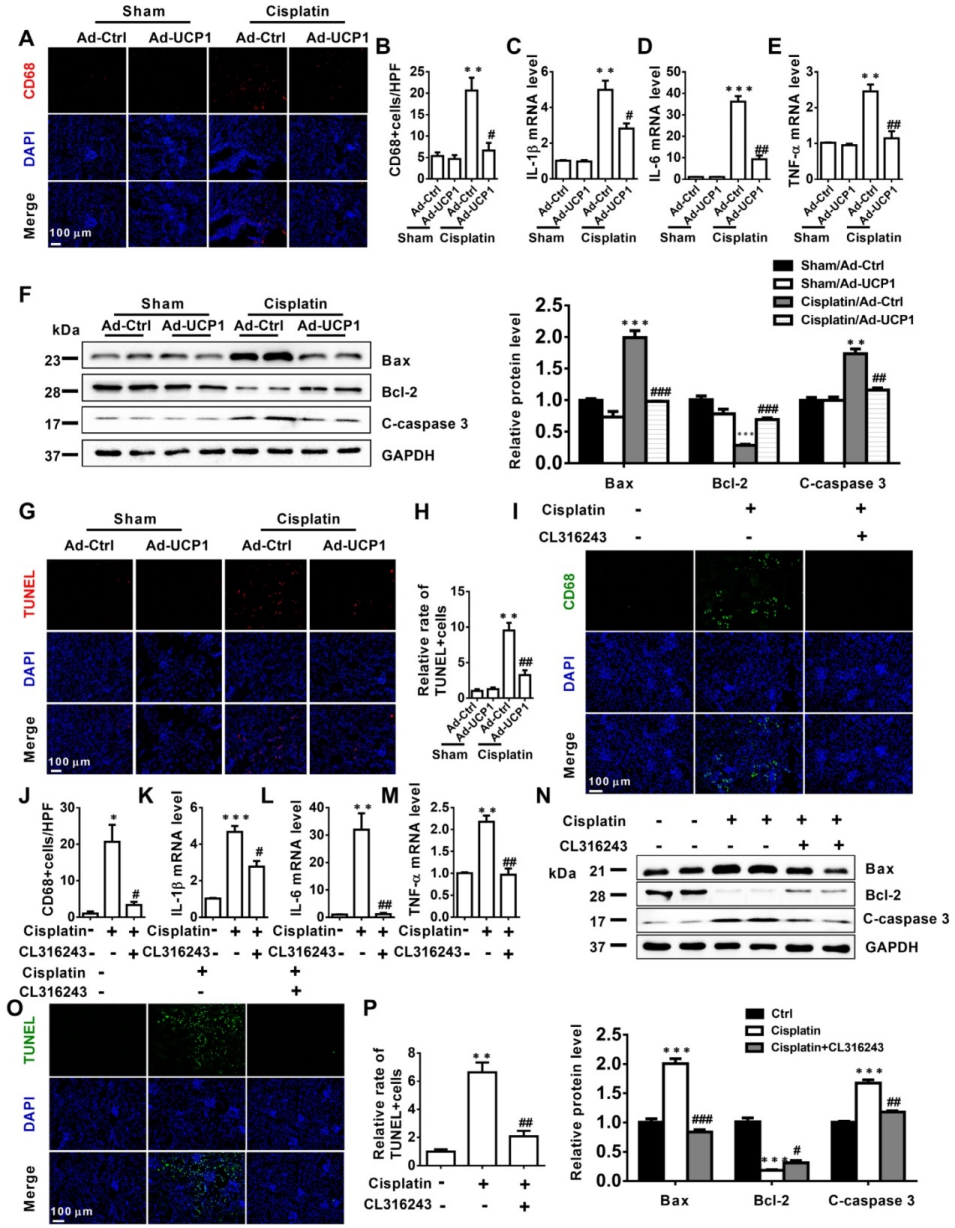
** Upregulation of UCP1 to relieve lipid accumulation in AKI can significantly alleviate inflammation and apoptosis *in vivo*.** (A, B) Immunofluorescence images and corresponding quantifications of CD68 in AKI animal models induced by cisplatin and injected with control or UCP1-expressing adenovirus. (C-E) IL-1β, IL-6, and TNF-α mRNA levels from AKI animal models specified in A. (F) Western blot images and corresponding quantifications of Bax, Bcl-2, and cleaved caspase-3 from AKI animal models specified in A. (G, H) TUNEL assay fluorescence images and corresponding quantifications of AKI animal models specified in A. (I, J) Immunofluorescence images and corresponding quantifications of CD68 in AKI animal models induced by cisplatin and treated with CL316243. (K-M) IL-1β, IL-6, and TNF-α mRNA levels from AKI animal models specified in I. (N) Western blot images and corresponding quantifications of Bax, Bcl-2, and cleaved caspase-3 from AKI animal models specified in I. (O, P) TUNEL assay fluorescence images and corresponding quantifications of AKI animal models specified in I. Nuclei are counterstained with DAPI. ****p < 0.0001, ***p < 0.001, **p < 0.01, *p < 0.05 vs. control; # vs. cisplatin.

The above study had confirmed that *in vitro*, upregulation of UCP1 could inhibit the progression of AKI by inhibiting inflammation and apoptosis. In order to explore the effect *in vivo*, we tested the corresponding indicators in the model of renal multipoint injection. As shown in **Figure 6A**-**E**, inflammation indicators CD68, IL-1β, IL-6, TNF-α all decreased significantly with UCP1 upregulation. In terms of apoptosis, the apoptotic indicators Bax and C-caspase 3 also decreased, while Bcl-2 increased with the upregulation of UCP1 (**Figure 6F**). TUNEL fluorescent staining also directly proved the decrease in apoptosis caused by upregulation of UCP1 (**Figure 6G**-**H**). Similarly, we also confirmed the same trend of inflammation and apoptosis in an animal model using the UCP1 agonist CL316243 (**Figure 6I**-**P**).

### Upregulation of UCP1 to reduce lipid accumulation during AKI promotes autophagy through the AMPK/ULK1 pathway *in vitro*

**Figure 7:**
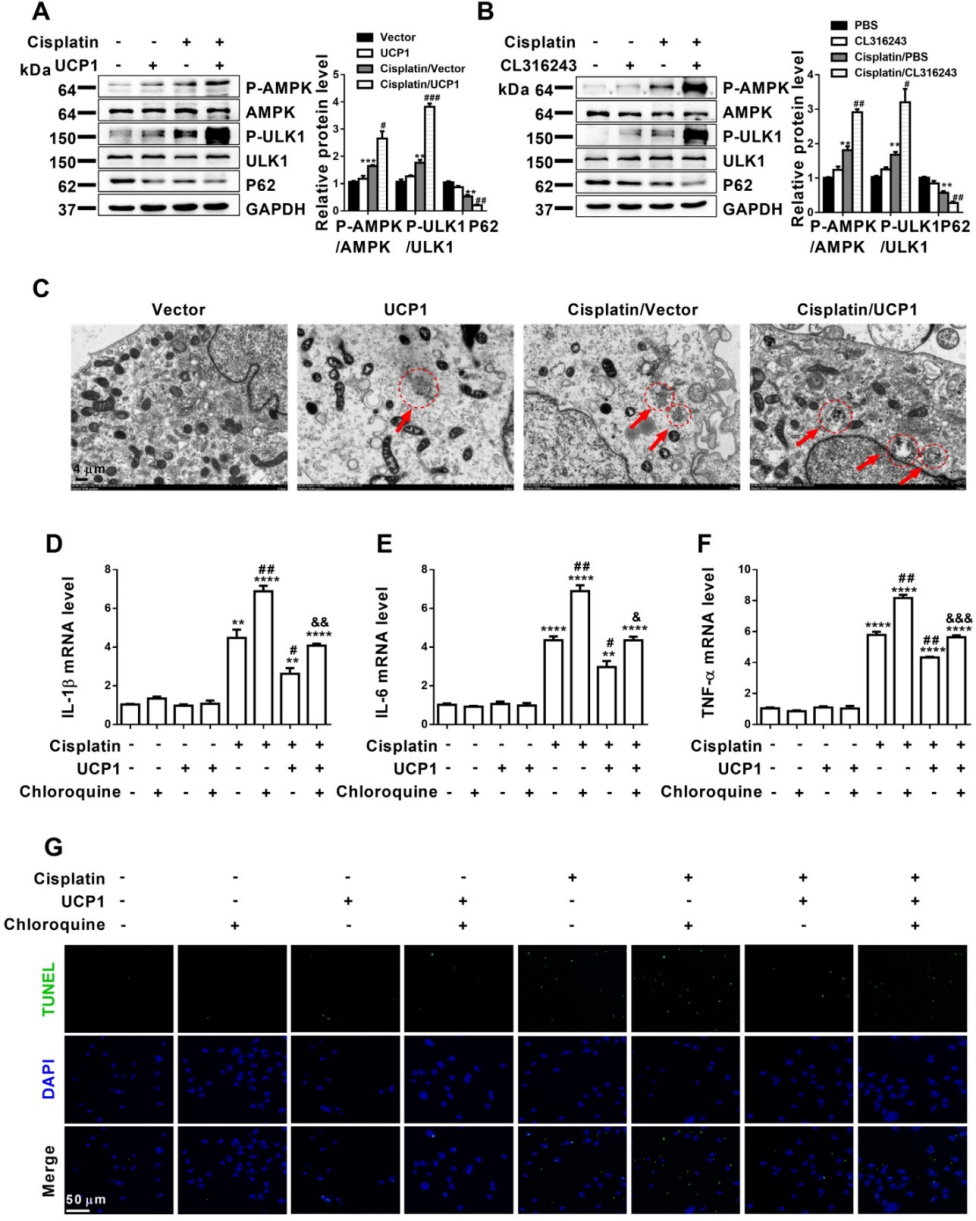
** Upregulation of UCP1 to reduce lipid accumulation in AKI promotes autophagy through the AMPK/ULK1 pathway *in vitro*.** (A, B) Western blot images and corresponding quantifications of AMPK/ULK1/autophagy pathway components in HK2 cells exposed to cisplatin with or without UCP1 overexpression. (C) Representative electron microscopic images showing autophagosomes of HK2 cells exposed to cisplatin with or without UCP1 overexpression. (D-F) IL-1β, IL-6, and TNF-α mRNA levels in HK2 cells with or without UCP1 overexpression and exposed or not to chloroquine. (G) TUNEL assay fluorescence images of HK2 cells with or without UCP1 overexpression and exposed or not to chloroquine. Nuclei are counterstained with DAPI. ****p < 0.0001, ***p < 0.001, **p < 0.01, *p < 0.05 vs. control; # vs. cisplatin; & vs. cisplatin with UCP1 overexpression.

Encouraged by the significant influence of lipid accumulation on AKI progression, we tried to explain the specific mechanism of lipid accumulation on the function of AKI. The regulation of lipid metabolism is highly related to mitochondria, which involves a variety of complex processes such as oxidative stress, mitochondrial stress, and autophagy 28, 29. Autophagy has been confirmed to play an important role in AKI, as it restores cell homeostasis and prevents AKI progression. It is a complex biological process, and its function involves many aspects such as cell activity, immunity and cell metabolism. Among them, lipid metabolism is closely related to autophagy 22. In particular, studies have reported that lipid metabolism in ccRCC was closely related to autophagy 13. Therefore, autophagy has become an important pathway of our concern. *In vitro* assessment of autophagy-related indicators in AKI cell models overexpressing UCP1 through lentivirus transfection or the UCP1 agonist CL316243 revealed significant activation of the AMPK/ULK1/autophagy pathway (**Figure 7A**-**B, Figure S8A**-**B**). Electron microscopy images also showed promoted autophagy after upregulating UCP1 to eliminate lipid accumulation in AKI (**Figure 7C**). Based on these findings, to verify the importance of autophagy in UCP1 function, we carried out functional recovery experiments. As shown in **Figure 7D**-**F**, inhibition of autophagy by chloroquine significantly reversed the decrease in inflammation indicators caused by UCP1 in cisplatin group. Similarly, TUNEL staining revealed that the use of chloroquine to inhibit autophagy strongly reversed the apoptosis inhibitory effect promoted by UCP1 (**Figure 7G**). These results showed that lipid accumulation played an important role in regulating cell function by acting on autophagy in AKI.

### Upregulation of UCP1 to reduce lipid accumulation during AKI promotes autophagy through the AMPK/ULK1 pathway *in vivo*

**Figure 8:**
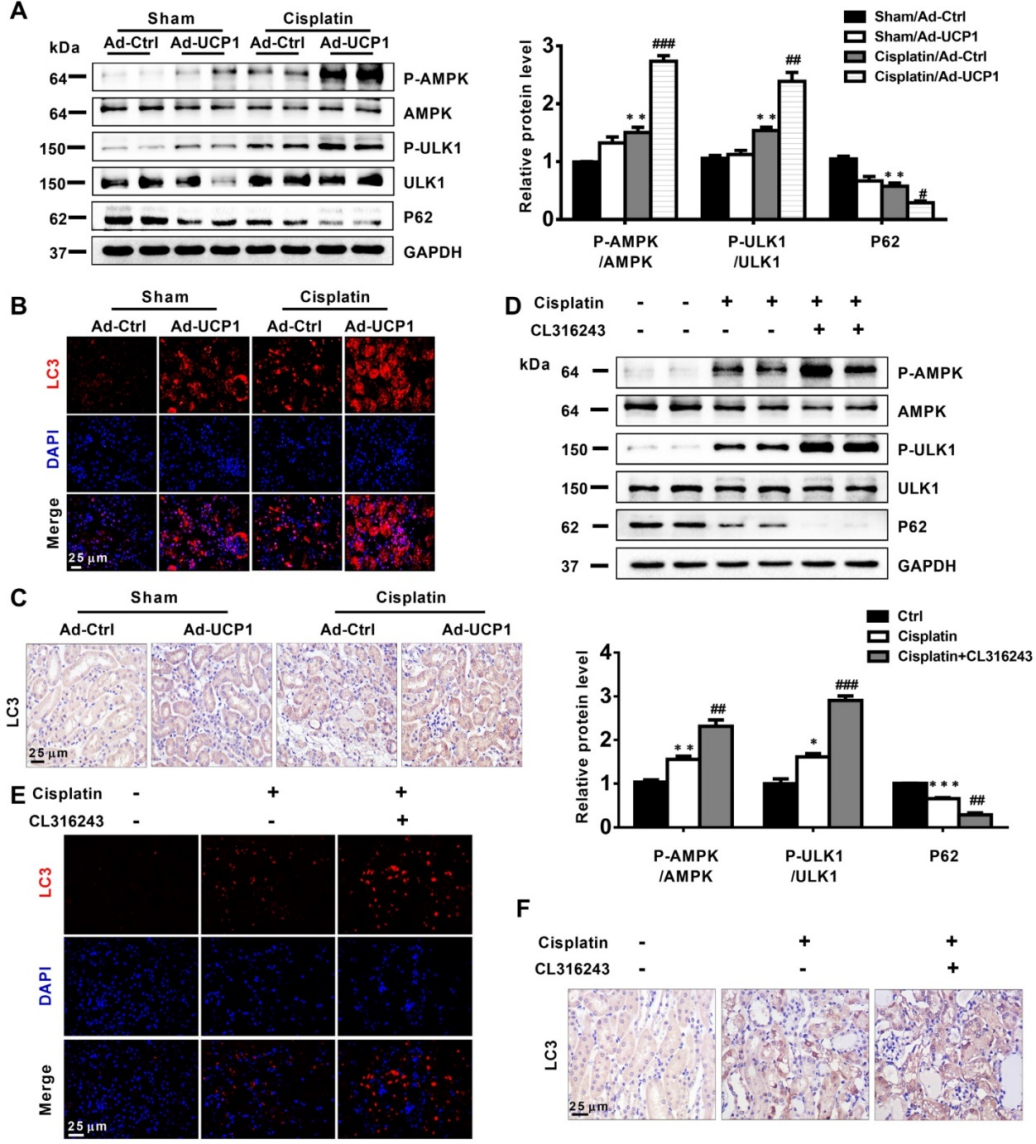
** Upregulation of UCP1 to reduce lipid accumulation in AKI promotes autophagy through the AMPK/ULK1 pathway *in vivo*.** (A) Western blot images and corresponding quantifications of AMPK/ULK1/autophagy pathway components in AKI animal models induced by cisplatin and injected with control or UCP1-expressing adenovirus. (B, C) Representative immunofluorescence images and immunohistochemistry staining against LC3 in AKI animal models specified in A. (D) Western blot images and corresponding quantifications of AMPK/ULK1 autophagy pathway components in animal AKI models induced by cisplatin and treated with CL316243. (E, F) Representative immunofluorescence images and immunohistochemistry staining against LC3 in AKI animal models specified in D. Nuclei are counterstained with DAPI. ****p < 0.0001, ***p < 0.001, **p < 0.01, *p < 0.05 vs. control; # vs. cisplatin.

Having discovered that UCP1 affected cellular functions in AKI by activating autophagy *in vitro*, we explored whether this occurred also *in vivo*. We detected autophagy-related indicators in animal models that increased UCP1 levels through renal multipoint injection to clear AKI lipid accumulation. The results showed that up-regulating UCP1 in animal models could also significantly activate AMPK/ULK1/autophagy pathway (**Figure 8A**), which was further confirmed by immunofluorescence and immunohistochemistry analyses (**Figure 8B**-**C**). Finally, the AMPK/ULK1/autophagy pathway was also significantly activated after upregulating UCP1 by CL316243 treatment (**Figure 8D**-**F**).

**Figure 9:**
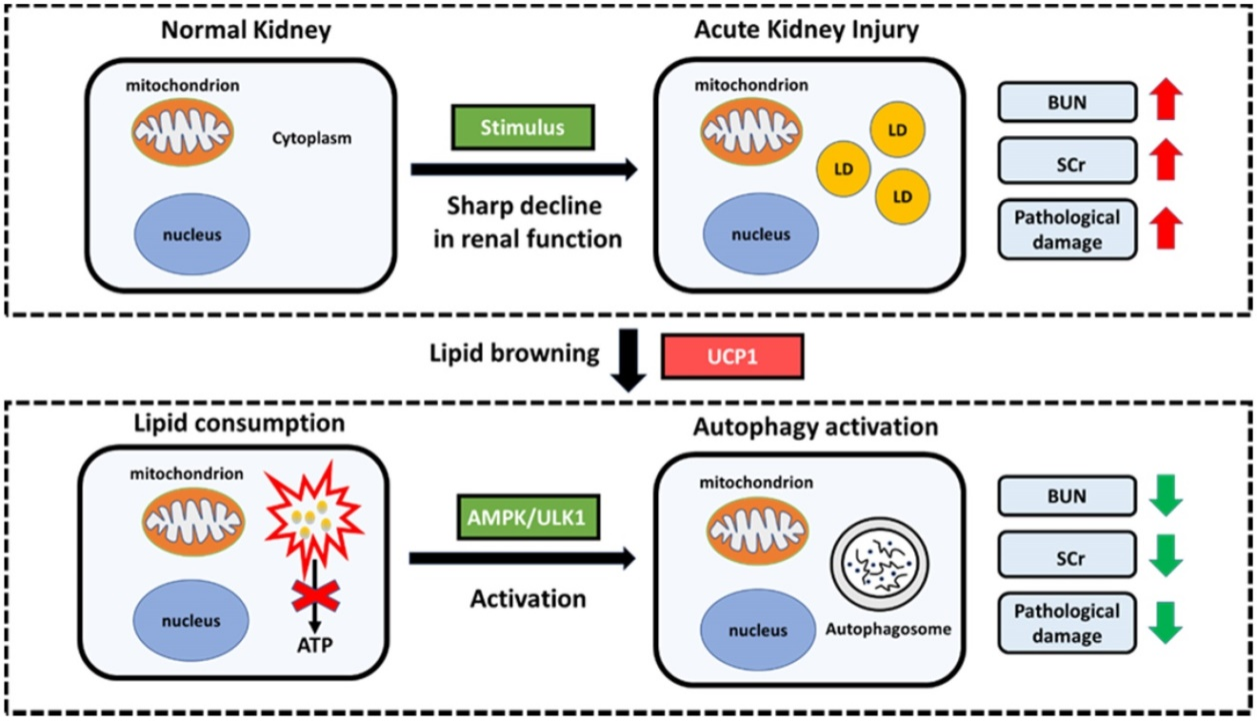
Proposed model illustrating the function and mechanism of lipid metabolism in AKI. Elevated lipid accumulation occurs in the renal tubules of AKI patients. UCP1 can eliminate abnormally accumulated lipids through lipid browning. UCP1-induced lipid removal activates AMPK/ULK1 pathway-mediated autophagy, thereby reducing the inflammatory response and apoptosis of tubule cells, ultimately recovering kidney function. LD, lipid droplets.

In summary, our research has constructed a model with significant lipid accumulation in AKI, and such accumulated lipids are negatively correlated with UCP1. By up-regulating UCP1 to clear the accumulated lipids in AKI, the AMPK/ULK1/autophagy pathway can be activated to inhibit disease progression (**Figure 9**).

## Discussion

AKI is a serious clinical emergency with a poor prognosis and a serious threat to the life and health of patients. At present, the treatment of AKI is mainly based on renal replacement therapy and symptomatic supportive treatment 2. Although the prognosis of the patient has been improved to a large extent, it still cannot fundamentally restore the normal function of the damaged renal cells, so the curative effect remains limited 3. Therefore, current efforts are focused on restoring renal function at the cellular level in AKI. Our research clarified the new mechanism of AKI from the perspective of lipid metabolism for the first time, discovered the differential expression of UCP1 in AKI, and clarified the significance of UCP1/AMPK/ULK1/autophagy pathway, which provides a new direction for the research of AKI.

In recent years, abnormal metabolism has been paid more and more attention by scientists. It has been proved to be related to the occurrence and development of numerous diseases, and even was a key step in the progression of some tumors, diabetes, obesity, and cardiovascular diseases 5. The occurrence of metabolic abnormalities is very complicated, which involves changes in glucose, lipid, and glutamine metabolism, each of which has its own characteristics and commonalities 5. Besides affecting nutrient cycling, metabolic alterations also affect cell growth, apoptosis, immunity and other key cellular functions 30.

The kidney is an organ highly related to overall body metabolism 31 through regulation of metabolites homeostasis and the elevated metabolic state of its cells 31, 32. Not surprisingly, metabolic abnormalities are closely related to kidney diseases. Tumors of kidney origin present a strong metabolic component, especially ccRCC, which is considered a metabolic disease 33. Similarly, non-neoplastic nephropathy is also found to be highly related to metabolic abnormalities. We know that hyperlipidemia is closely related to kidney diseases 34. It is a common clinical manifestation of many primary or secondary kidney diseases, and itself participates in the occurrence and development of kidney diseases 34. In 1982, Moorhead proposed that hyperlipidemia was an independent pathogenic factor in the development of glomerulosclerosis 35. In normal rats, high cholesterol diet could lead to kidney lipid deposition, and then develop into focal segmental glomerulosclerosis (FSGS) 36. It is found that the increase of cholesterol content and abnormal accumulation of phospholipids and fatty acids in renal cortex were closely related to glomerular interstitial diseases 37. Patients with renal lipoprotein deposition had more severe proteinuria and higher tendency of glomerulosclerosis than patients without renal lipoprotein deposition 38.

AKI is accompanied by abnormal glucose, amino acid and lipid metabolism 39. In contrast to studies on the role of glucose and amino acid metabolism in AKI, no systematic investigation on lipid metabolism in AKI has been carried out so far. Our study systematically analyzed the differences of lipid components in AKI for the first time, clarified that there was significant lipid accumulation in AKI. In addition, the accumulation of lipids in AKI was highly positively correlated with the severity of the disease, and reducing this type of lipid accumulation could significantly alleviate the progress of AKI. Abnormal lipid metabolism has been proved to be both a factor leading to disease occurrence and an important factor leading to disease progression in many conditions 40-42. For example, in patients with diabetes or obesity, the accumulation of lipids in the body could induce the disease occurrence, while abnormal glucose metabolism also had an important impact on lipid accumulation, thereby further accelerating the progression of the disease 40, 41. In previous studies on kidney diseases, it is found that there was significant lipid accumulation in glomerulosclerotic diseases, and abnormal lipid metabolism further aggravated proteinuria and glomerulosclerosis 35. In AKI, we have similar findings, that is, abnormal lipid metabolism has appeared in the early stage of AKI. Therefore, we believe that lipid accumulation may be a cause of renal tubular damage. As the disease worsening, the accumulation of lipids gradually increases, and it can also be considered as an important factor in disease progression.

The process of lipid metabolism is very complex, which involves a special phenomenon named lipid browning. Body lipids can be roughly divided into WAT and BAT. WAT is used for energy storage, while BAT is used for heat production. The process of lipid browning is the transformation from WAT to BAT, which makes the lipids more easily consumed without producing ATP 11. UCP1 is the most direct and important mediator of lipid browning owing to its uncoupling effect 9. So far, most of the studies on lipid browning have focused on obesity and diabetes 10. Until recently, it has been reported that lipid browning can inhibit tumor progression by activating lipid browning in ccRCC, which is defined as “tumor slimming” 14. In this study, we found the differential expression of UCP1 in AKI, and found that it was highly dependent on lipids. Upregulation of UCP1 could significantly eliminate lipid accumulation in AKI and inhibited its progression. The same effect could also be achieved through the use of UCP1 agonists. Our findings provide a new direction for the treatment of AKI, namely targeted lipid metabolism therapy, particularly with respect to UCP1. Because UCP1 is highly related to obesity, a large number of specific weight loss drugs have been developed 8, 43, 44. Therefore, whether these drugs can assist the treatment of AKI has become a new possibility.

Autophagy is a process in which organelles and proteins which need to be degraded in the cytoplasm are wrapped by double membrane and fused with lysosome to form autophagy lysosome to digest the substances in autophagy body 15. It has a wide range of functions, which mainly involves the regulation of programmed cell death, inflammation and other biological processes 16. Autophagy is a double-edged sword. In general, it can maintain the homeostasis of normal cellular environment and inhibit the occurrence of malignant transformation. However, when the stimulation is too strong, autophagy can play the opposite role 17. Previous studies have found that autophagy in kidney tissue was significantly activated after stimulation by cisplatin, and the kidney injury was aggravated after proximal tubule-specific autophagy deficiency 45. At the same time, autophagy induction via rapamycin reduced cisplatin induced apoptosis and renal damage 45. Therefore, the activation of autophagy in kidney tissue seems to play a protective role in acute kidney injury. AMPK, an energy-sensitive kinase, is the key molecule in the regulation of bioenergy metabolism 46. The expression of phosphorylated AMPK in kidney tissue of acute kidney injury is significantly increased, moreover, the activation of AMPK could induce autophagy by inhibiting mTOR signaling pathway 19, 47. Our study found that clearance of lipid accumulation in AKI could significantly activate autophagy by activating AMPK/ULK1 pathway, thus inhibiting AKI progression. It is worth noting that there are active and feedback activation in the regulation of autophagy 48. In our study, inhibition of AMPK/ULK1/autophagy pathway reversed the effect of UCP1 on inflammation and apoptosis. Therefore, based on our experimental results, we believe that autophagy was involved in the regulation of UCP1 on AKI in the way of active activation. This also provides a new idea for the treatment of AKI. In addition to targeted lipid therapy, autophagy targeted drugs are likely to play an auxiliary role.

In general, this is the first study to systematically analyzed the differences in lipid composition in AKI, and clarified lipid accumulation as a salient marker of the disease. At the same time, the study highlights the close relationship between abnormal lipid accumulation and UCP1 in AKI. UCP1-dependent lipid clearance could activate AMPK/ULK1 pathway to elevate autophagy level, inhibit inflammation and apoptosis, and delay the progression of AKI. This study identifies new targets for the treatment of AKI, and provides the possibility for the development of new drugs and combination therapy.

## Conclusions

The present findings suggest that lipid accumulation in AKI is directly regulated by UCP1, which can activate cell autophagy and thus significantly inhibit disease progression. It will provide new ideas and targets for the treatment of AKI.

## Supplementary Material

Supplementary figures.

## Figures and Tables

**Table 1:** List of primers for PCR of mice

Primer	Sequence
**GAPDH**	
Forward	5'-ATGGTGAAGGTCGGTGTGAA-3'
Reverse	5'-TGGAAGATGGTGATGGGCTT-3'
**IL-1β**	
Forward	5'- TCAGGCAGGCAGTATCACTC-3'
Reverse	5'- AGCTCATATGGGTCCGACAG-3'
**IL-6**	
Forward	5'- TTCTTGGGACTGATGCTGGT-3'
Reverse	5'- CAAGTGCATCATCGTTGTTCA-3'
**TNF-α**	
Forward	5'- GTGCCTATGTCTCAGCCTCT-3'
Reverse	5'- TGGTTTGTGAGTGTGAGGGT-3'

**Table 2:** List of primers for PCR of human

Primer	Sequence
**GAPDH**	
Forward	5'- GAGTCAACGGATTTGGTCGT-3'
Reverse	5'- GACAAGCTTCCCGTTCTCAG-3'
**IL-1β**	
Forward	5'- GCTGAGGAAGATGCTGGTTC-3'
Reverse	5'- TCCATATCCTGTCCCTGGAG-3'
**IL-6**	
Forward	5'- AGGAGACTTGCCTGGTGAAA-3'
Reverse	5'- CAGGGGTGGTTATTGCATCT-3'
**TNF-α**	
Forward	5'- ATCAGAGGGCCTGTACCTCA-3'
Reverse	5'- GGAAGACCCCTCCCAGATAG-3'

## Abbreviations

UCP1: uncoupling protein 1
AKI: acute kidney injury
SCr: serum creatinine
BUN: urea nitrogen
IL-1β: interleukin-1β
IL-6: interleukin-6
TNF-α: tumor necrosis factor-alpha
ccRCC: renal cell carcinoma
LD: lipid droplets
