# Supplementary material for: Relieving lipid accumulation through UCP1 suppresses the progression of acute kidney injury by promoting the AMPK/ULK1/autophagy pathway
Source: Theranostics. 2021 Mar 4;11(10):4637–54. doi: 10.7150/thno.56082 (PMC7978316; doi:10.7150/thno.56082)

Supplementary figure legends

Supplemental Fig. 1 PCA method was used to observe the overall distribution trend between the control and cisplatin groups.

Supplemental Fig. 2 Cluster analysis chart based on lipid metabolomics results of the control group and cisplatin-induced AKI group (n = 8).

Supplemental Fig. 3 Lipid maps annotation based on lipid metabolomics sequencing (n = 8).

Supplemental Fig. 4 Enrichment analysis of KEGG-related pathways based on lipid metabolomics results (n = 8).

Supplemental Fig. 5 The degree of renal injury increased gradually with the prolongation of cisplatin stimulation time. (A, B) BUN and SCr levels from animal models treated with cisplatin for 1, 2, 4 days (n = 5). (C) Western blot images and corresponding quantifications of NGAL in HK2 cells exposed to cisplatin for 3, 6, 12,24h. **** $p < 0.0001$, *** $p < 0.001$, ** $p < 0.01$, * $p < 0.05$.

Supplemental Fig. 6 UCP1 and lipid droplets were mainly located in renal tubules. (A) Representative confocal microscopic images of lectin and UCP1 in the kidneys of control mice. (B) Representative confocal microscopic images of AQP1 and UCP1 in the kidneys of control mice. (C) Representative confocal microscopic images of AQP1 and BODIPY dye in the

kidneys of mice treated with cisplatin. Nuclei are counterstained with DAPI.

Supplemental Fig. 7 The content of triglyceride (TG) in renal tissues. (A, B) Specific administration methods of animal models. (C) Relative TG content (gprot/L) in AKI models induced by cisplatin with control or UCP1 adenovirus injection tested by TG assay kit. (D) Relative TG content (gprot/L) in AKI models induced by cisplatin with CL316243 treatment. **** $p < 0.0001$, *** $p < 0.001$, ** $p < 0.01$, * $p < 0.05$.

Supplemental Fig. 8 The relative protein level of LC3 in HK2 cells. (A, B) Western blot images and corresponding quantifications of LC3 in HK2 cells exposed to cisplatin with or without UCP1 overexpression. **** $p < 0.0001$, *** $p < 0.001$, ** $p < 0.01$, * $p < 0.05$.

Supplemental Fig. 1

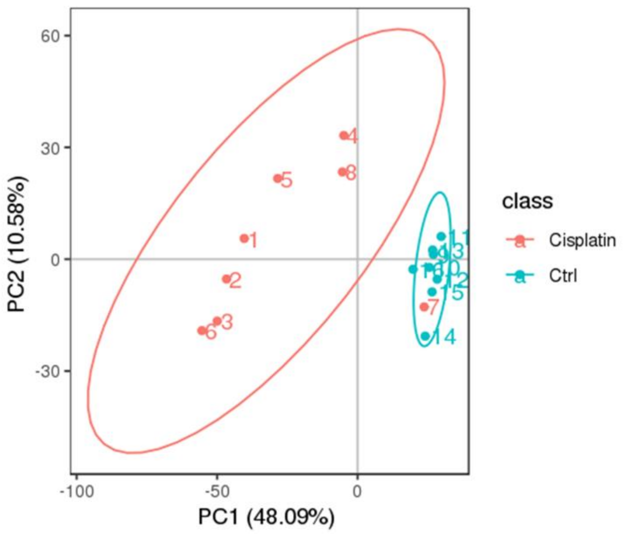

Supplemental Fig. 2

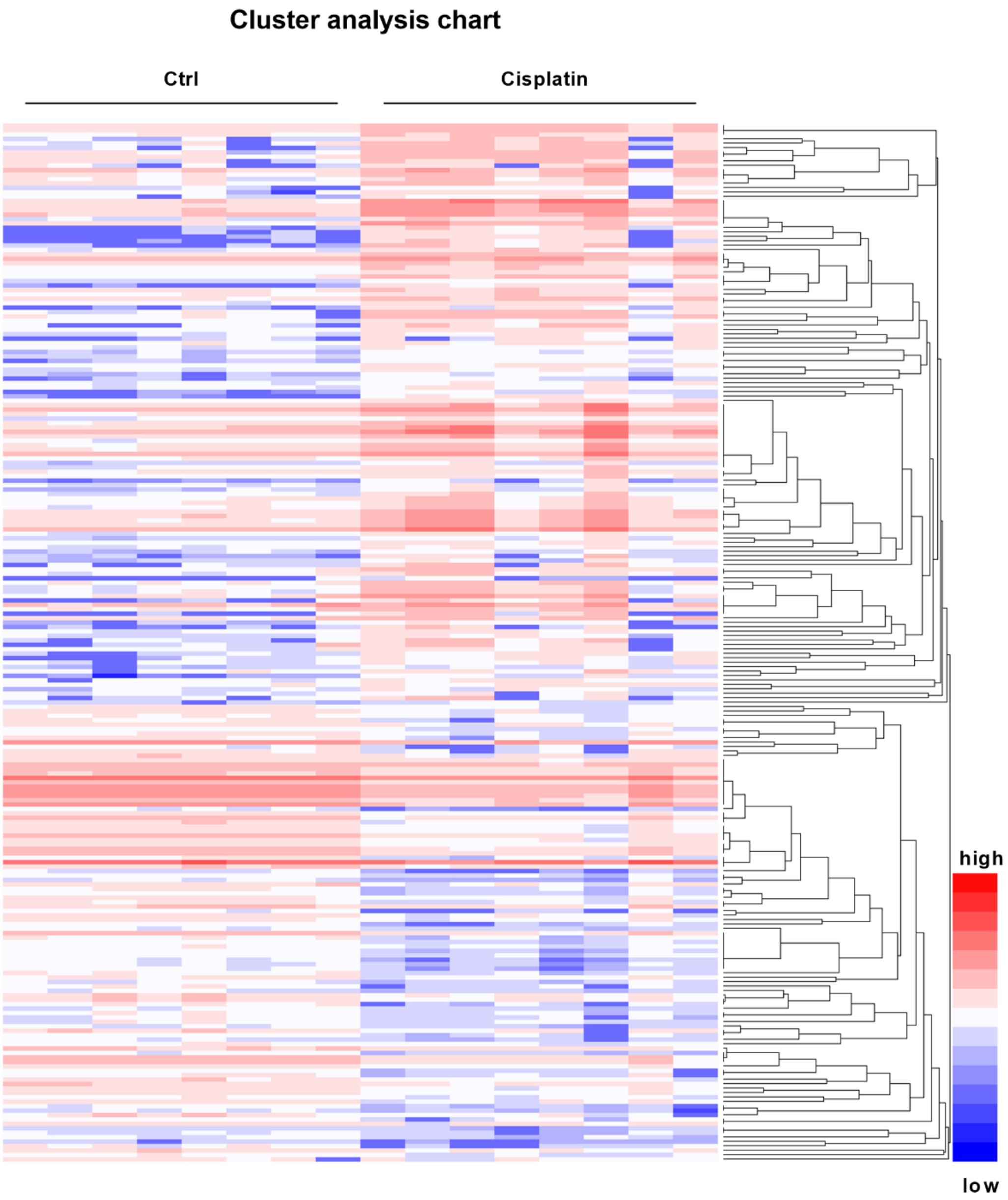

Supplemental Fig. 3

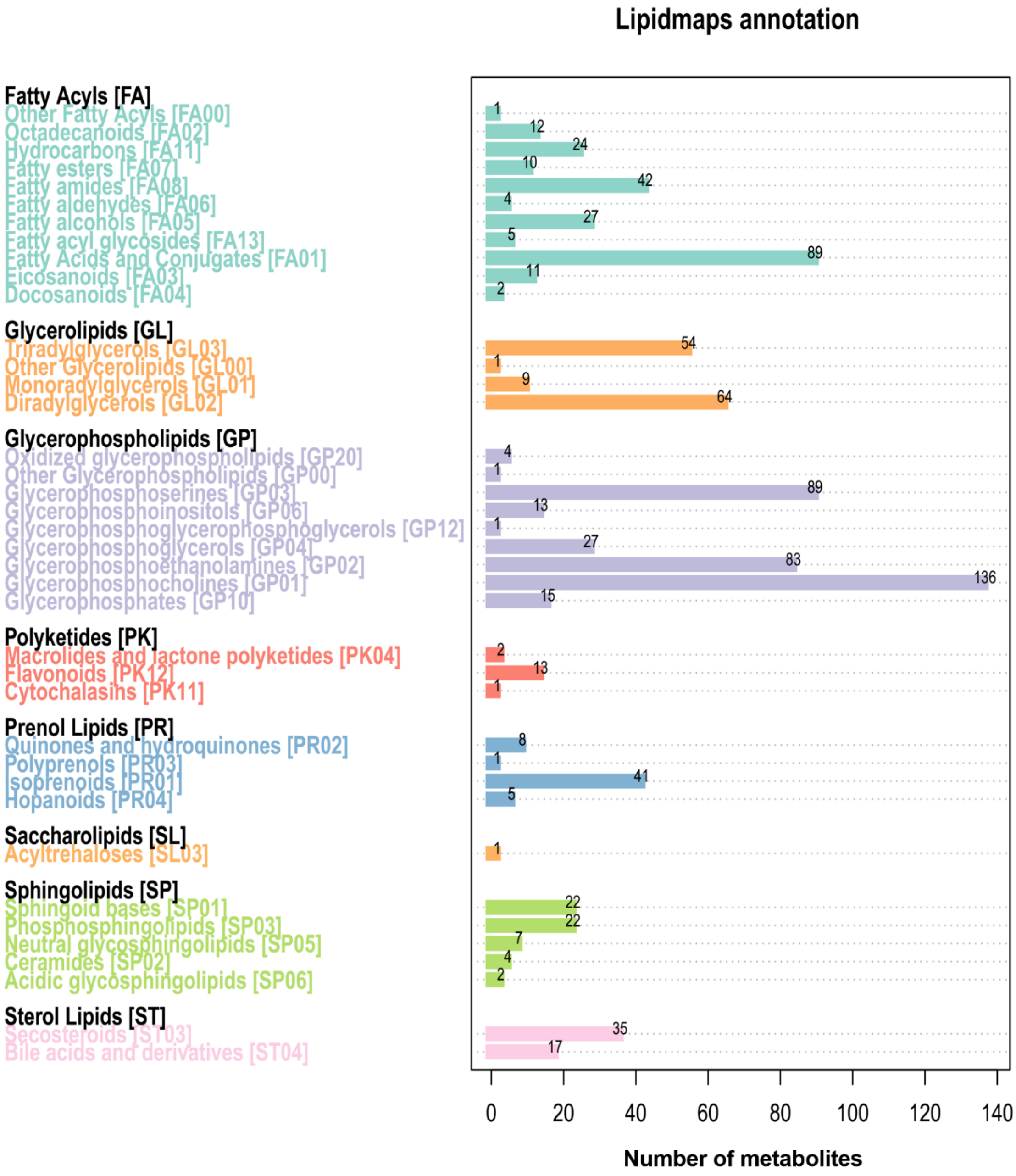

Supplemental Fig. 4

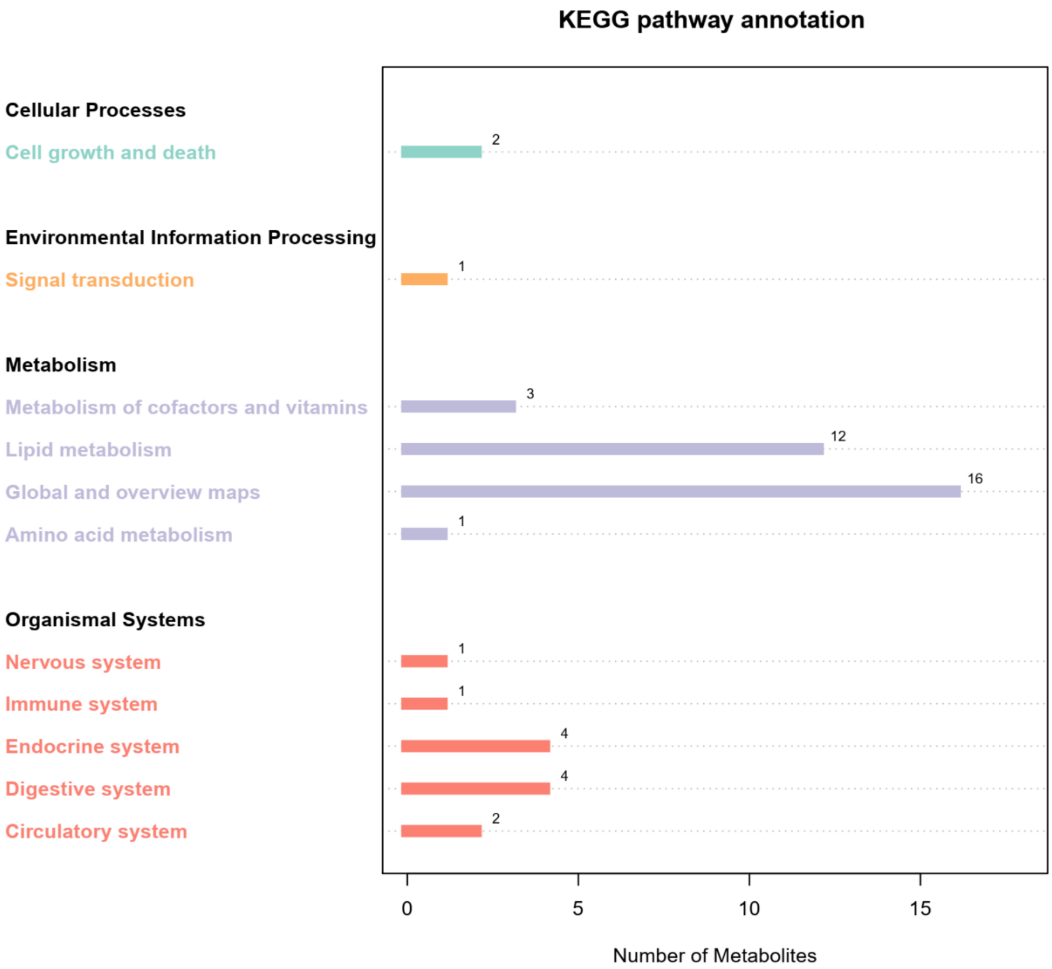

Supplemental Fig. 5

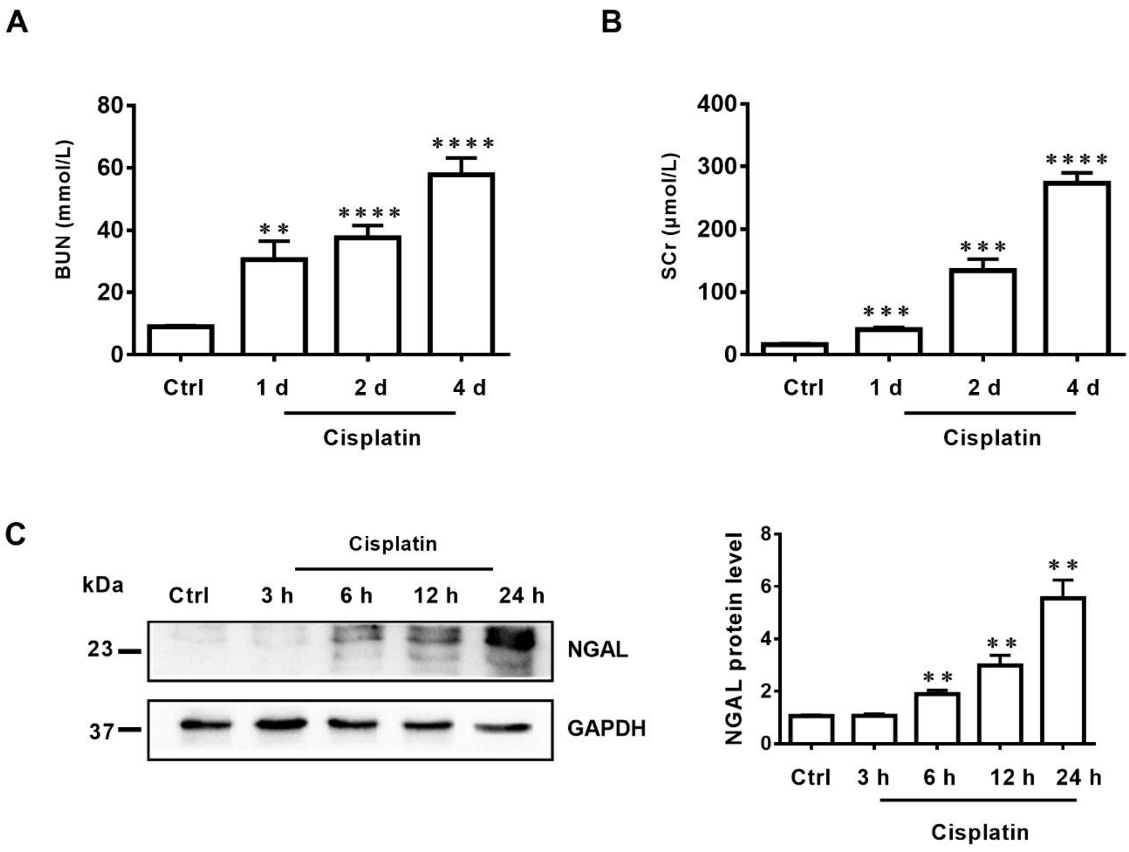

Supplemental Fig. 6

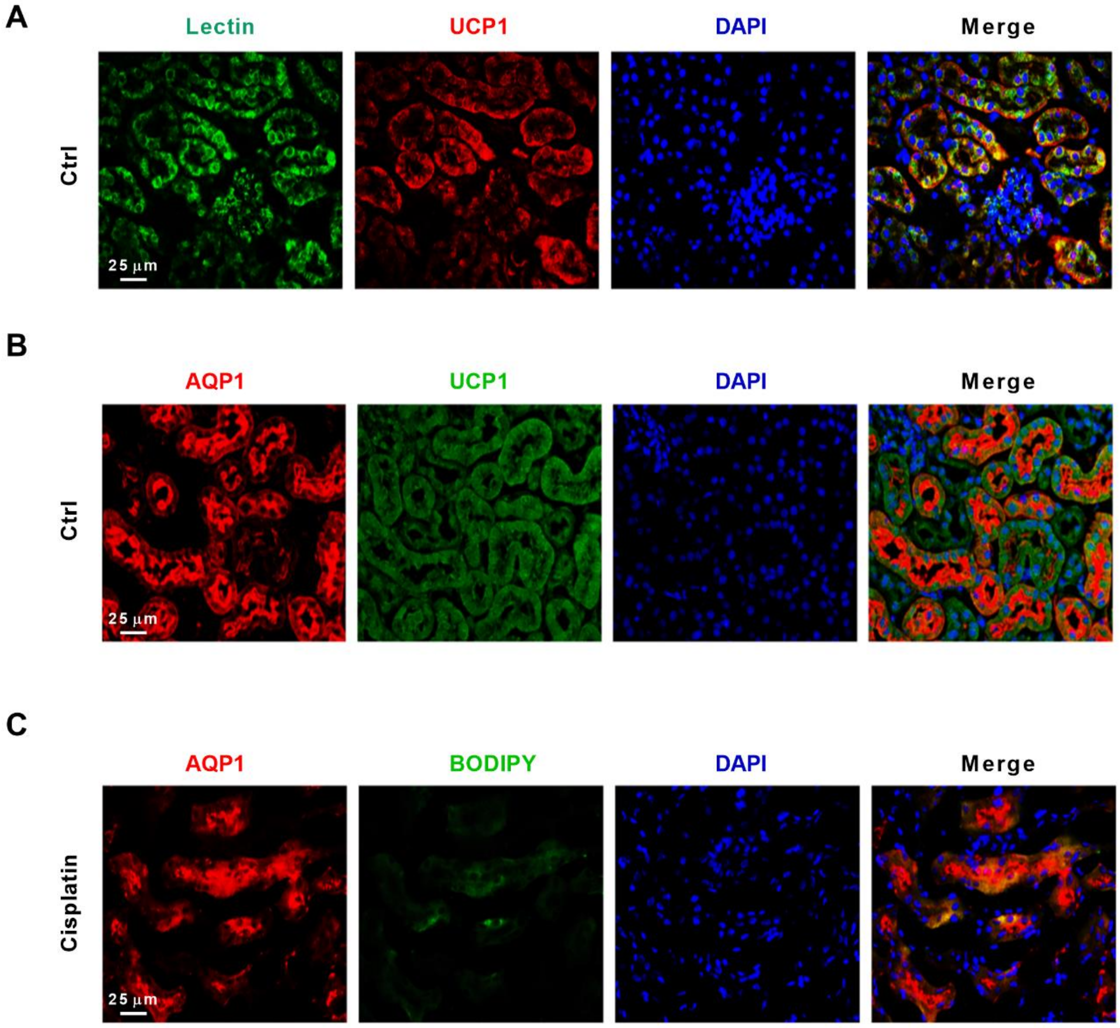

Supplemental Fig. 7

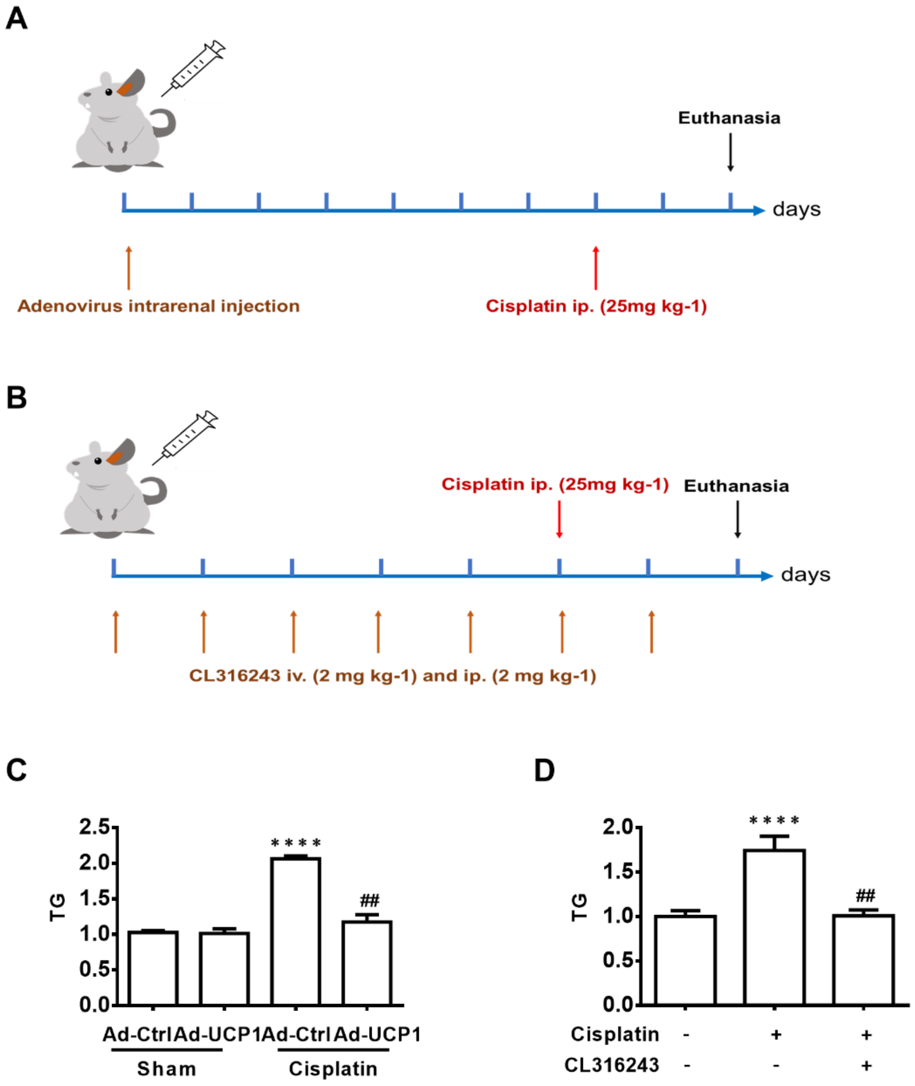

Supplemental Fig. 8

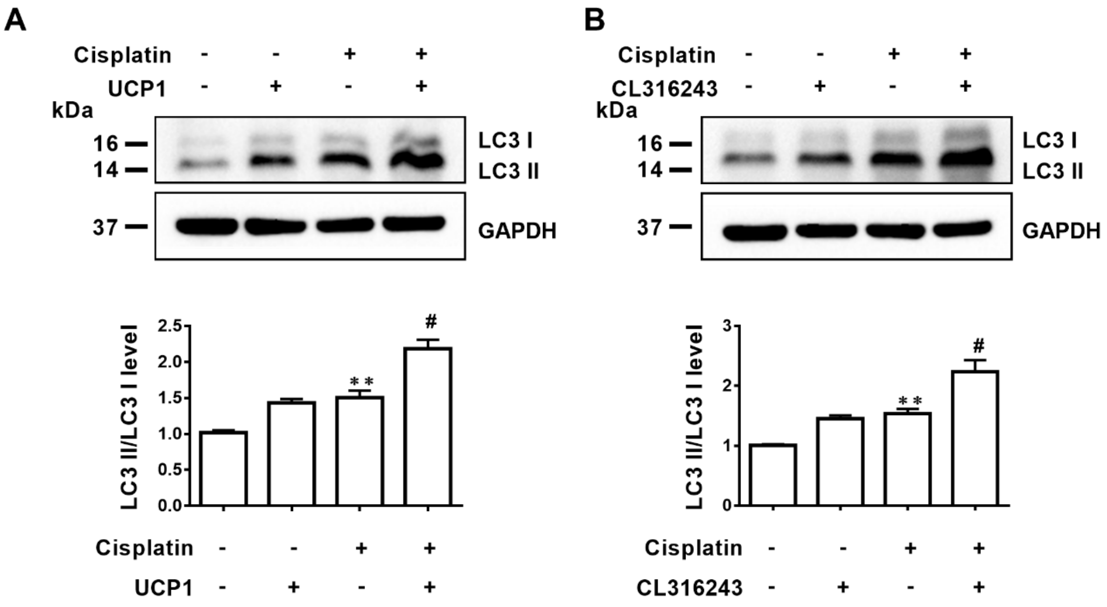

Supplement: Supplementary file 1 — Supplementary figures. [file thnov11p4637s1.pdf]
